# IgG4-related aortitis mimicking acute aortic and coronary syndromes, multimodality imaging–pathology correlation: a case report

**DOI:** 10.3389/fcvm.2026.1819638

**Published:** 2026-06-04

**Authors:** Laura Victoria Torres-Araujo, Valente Fernandez-Badillo, Antonio Jordan-Rios, Rodrigo Gopar-Nieto, Silvia Jimenez-Becerra, Jesus A. Cotes-Millan, Daniel Sierra Lara-Martinez, Benjamin I. Hernandez-Mejia, Moises Jimenez-Santos, Sergio A. Criales-Vera

**Affiliations:** 1 Inpatient Care Unit, National Institute of Cardiology Ignacio Chavez, Mexico City, Mexico; 2 CT Scanner Lomas Altas, Lomas Altas, Mexico City, Mexico; 3 Cardiology Resident, Department of Clinical Cardiology, National Institute of Cardiology Ignacio Chavez, Mexico City, Mexico; 4 Heart Failure Unit, National Institute of Cardiology Ignacio Chavez, Mexico City, Mexico; 5 Coronary Care Unit, National Institute of Cardiology Ignacio Chavez, Mexico City, Mexico; 6 Department of Pathology, National Institute of Cardiology Ignacio Chavez, Mexico City, Mexico; 7 Department of Cardiovascular Imaging, National Institute of Cardiology Ignacio Chavez, Mexico City, Mexico; 8 Department of Cardiothoracic Surgery, National Institute of Cardiology Ignacio Chavez, Mexico City, Mexico; 9 CT Scanner México, Mexico City, Mexico

**Keywords:** acute aortic syndrome, acute coronary sydrome, aortitis, chest pain, igG4, igG4 - related disease, NSTEMI

## Abstract

**Background:**

IgG4-related aortitis is an uncommon inflammatory condition that can closely mimic both acute aortic and coronary syndromes, posing a major diagnostic challenge in patients presenting with chest pain.

**Case summary:**

A 64-year-old man with prior non–ST-elevation myocardial infarction (NSTEMI) and persistent angina presented with abrupt-onset, severe retrosternal chest pain and dynamic lateral ST-segment depression. Rising high-sensitivity troponin supported a working diagnosis of high-risk NSTEMI. During observation, he developed acute respiratory failure and hemodynamic collapse. Echocardiography revealed a large circumferential pericardial effusion with reduced left ventricular ejection fraction, without classic tamponade physiology. Contrast-enhanced computed tomography demonstrated a crescentic ascending aortic wall thickening consistent with Stanford type A intramural hematoma extending into the arch vessels. A pericardial window drained 550 mL of serohemorrhagic fluid, with transient improvement, but recurrent instability prompted emergent ascending aortic replacement and coronary bypass grafting. Despite maximal support, the patient died intraoperatively. Histopathological analysis revealed dense lymphoplasmacytic infiltration rich in IgG4-positive plasma cells and storiform fibrosis, confirming IgG4-related aortitis.

**Discussion:**

This case highlights the ability of IgG4-related aortitis to mimic both intramural hematoma and acute coronary syndromes, illustrating a critical diagnostic blind spot in acute cardiovascular care, particularly when imaging findings, clinical presentation, and intraoperative observations are discordant.

## Graphical Abstract

**Figure:**
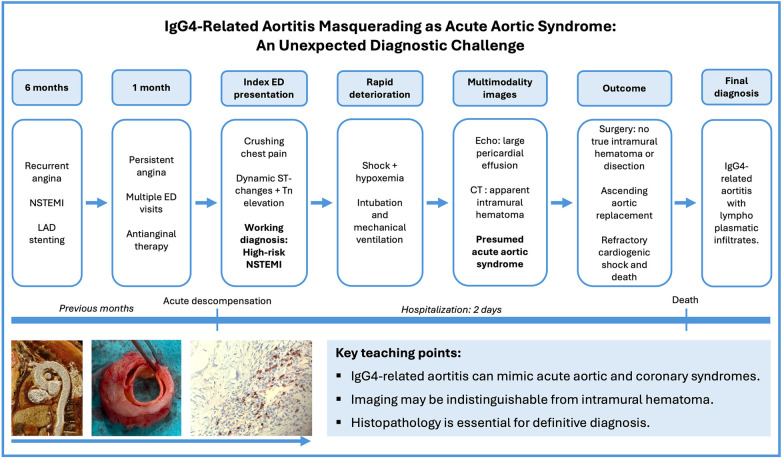
IgG4-related aortitis masquerading as acute aortic and coronary syndromes: a diagnostic challenge

## Introduction

IgG4-related disease (IgG4-RD) is a systemic fibroinflammatory condition characterized by tumefactive lesions, dense lymphoplasmacytic infiltration rich in IgG4-positive plasma cells, storiform fibrosis, and, in some cases, obliterative phlebitis (1, 2). Although pancreatic and salivary gland involvement predominate, vascular manifestations are increasingly recognized. Large-vessel involvement occurs in approximately 10%–30% of patients, most commonly affecting the abdominal aorta and retroperitoneum (3, 4), whereas thoracic involvement is less frequent and ascending aortic disease accounts for fewer than 5%–10% of vascular cases, contributing to its under-recognition in acute cardiovascular settings (4, 5).

A major diagnostic challenge is the ability of IgG4-related aortitis to closely mimic acute aortic syndromes (AAS), particularly intramural hematoma (IMH), due to overlapping imaging features such as concentric wall thickening and intermediate attenuation on computed tomography (CT) (6). In patients presenting with acute chest pain, this radiologic overlap may lead to diagnostic misclassification and prompt emergent surgical intervention. Although multimodality imaging plays a central role in the evaluation of acute cardiovascular presentations, it may be insufficient to reliably distinguish inflammatory from structural aortic pathology in hyperacute settings.

Current diagnostic algorithms for acute aortic syndromes do not incorporate inflammatory aortitides such as IgG4-RD, and clinical suspicion is often low, particularly in unstable patients. As a result, diagnosis is frequently established only after surgical intervention through histopathological examination, which remains the gold standard when imaging and clinical findings are inconclusive (2, 7, 8). We report a fatal case of IgG4-related aortitis that simultaneously mimicked acute coronary and aortic syndromes, with comprehensive multimodality imaging, intraoperative correlation, and definitive histopathological confirmation, highlighting a critical diagnostic blind spot in acute cardiovascular care.

## Case description

A 64-year-old man presented to the emergency department with the most severe episode to date of crushing retrosternal chest pain radiating to the mandible and both shoulders. The pain began abruptly at rest, was rated 10/10 in intensity, and was accompanied by dyspnea, orthopnea, and marked anxiety. On arrival, he was alert but visibly distressed. Blood pressure was 118/72 mmHg, heart rate 92 bpm, respiratory rate 22 breaths/min, and oxygen saturation 96% on room air. Cardiopulmonary and vascular examinations were unremarkable, with no pulse deficits or differential limb pressures. The presentation was most consistent with unstable angina or high-risk non–ST-elevation myocardial infarction (NSTEMI).

Six months earlier, he had developed intermittent exertional chest pain and was diagnosed with NSTEMI at an outside hospital. Coronary angiography revealed chronic diffuse left anterior descending artery (LAD) disease, a 50% proximal right coronary artery (RCA) stenosis, and mild disease in the remaining vessels. Two drug-eluting stents were placed in the proximal and mid-LAD. He was discharged on dual antiplatelet therapy and statin treatment. Persistent angina led to escalation of antianginal therapy at our institution, including beta-blockers, nitrates, calcium channel blockers, ivabradine, trimetazidine, and intensified lipid-lowering therapy. He returned to the emergency department twice in the preceding month with chest pain but no biomarker elevation. His history was otherwise notable for minimal tobacco exposure (1.5 pack-years) and sulfonamide allergy.

Initial electrocardiography showed QS complexes in V1–V2 and dynamic lateral ST-segment depression (Supplementary Figure 1). High-sensitivity troponin T was 55 ng/L (reference <14 ng/L), rising to 100 ng/L at 2 h. C-reactive protein was 14.3 mg/L (reference <1 mg/L), and NT-proBNP was 1,492 pg/mL (reference value <300 pg/mL), consistent with systemic inflammation and hemodynamic stress. A high-risk NSTEMI was diagnosed, and the patient was admitted for urgent coronary evaluation within 24 h.

**Figure 1:**
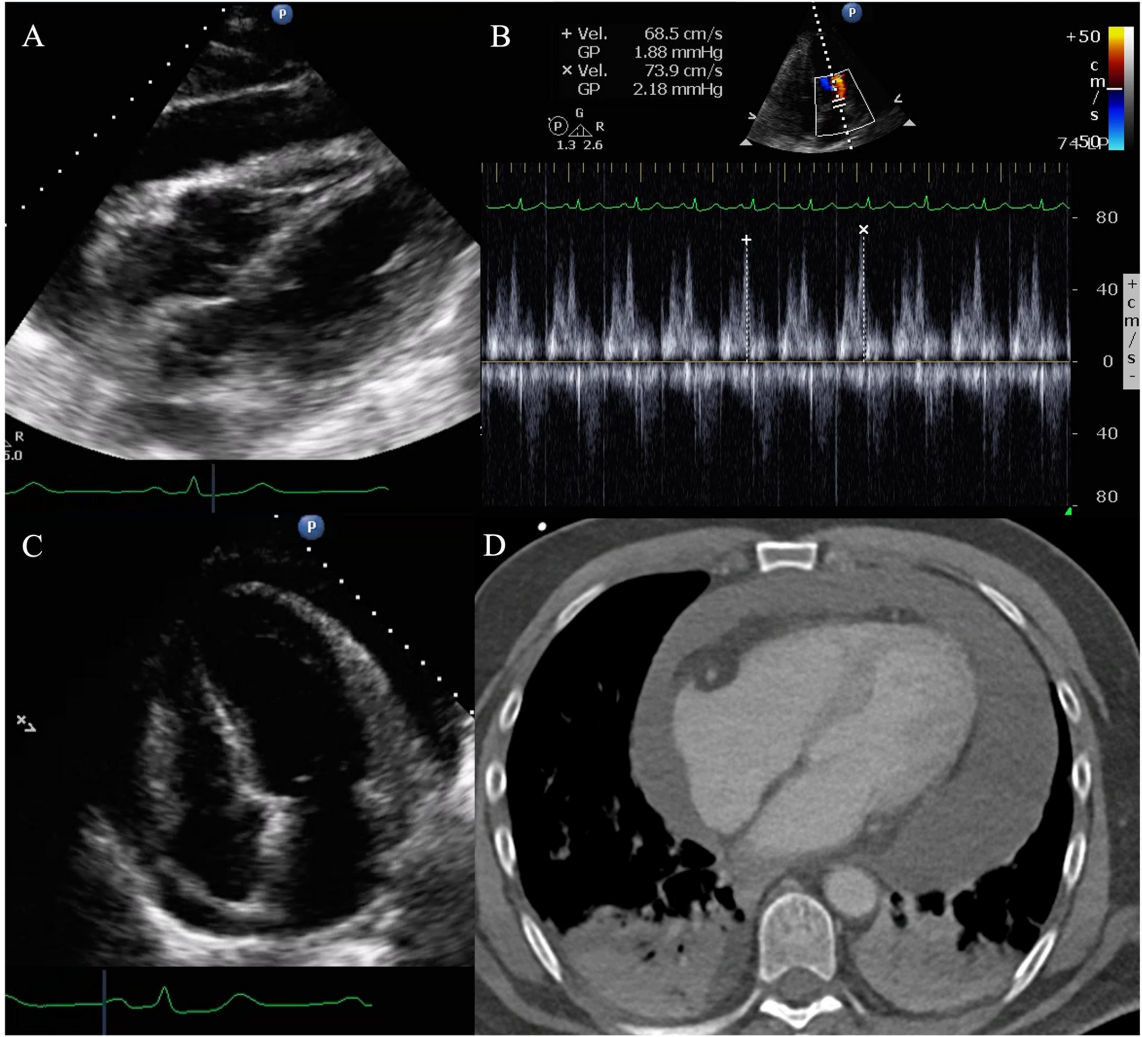
**Pericardial effusion on multimodality imaging. (A)** Subxiphoid transthoracic echocardiography shows a large circumferential pericardial effusion with reduced left ventricular systolic function. **(B)** Pulsed-wave Doppler of transmitral inflow demonstrates no significant respiratory variation. **(C)** Apical four-chamber view confirms a large pericardial effusion. **(D)** Contrast-enhanced chest computed tomography reveals a large pericardial effusion and concentric thickening of the ascending aortic wall, initially interpreted as an acute aortic syndrome.

**Figure 2:**
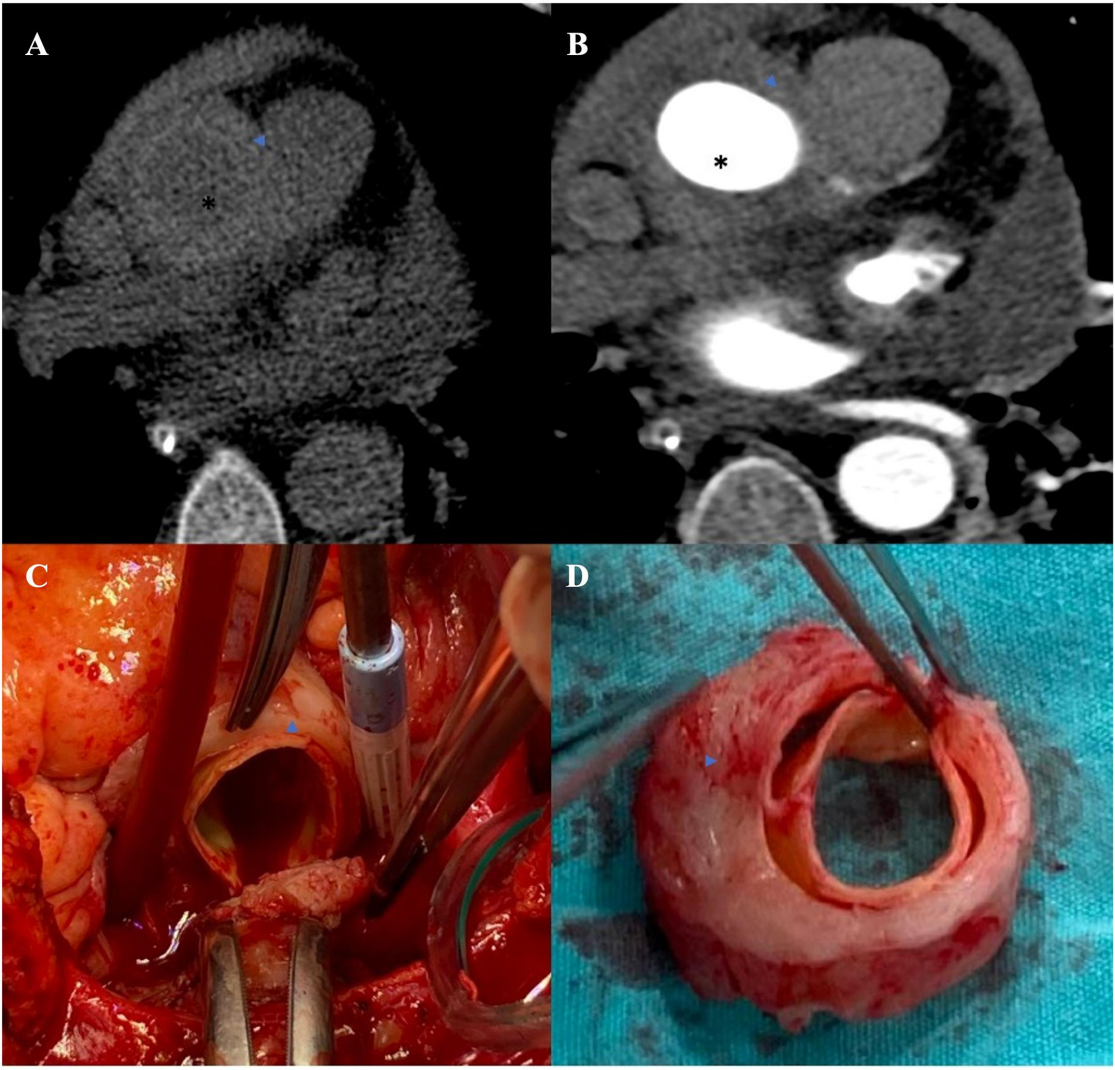
**Surgical and tomographic correlation. (A)** Unenhanced computed tomography image shows a crescentic area of high attenuation (blue arrowhead) along the walls of the ascending aorta (*). **(B)** Contrast-enhanced computed tomography image shows diffuse abnormal thickening of the thoracic aorta (blue arrowhead. **(C)** Intraoperative exposure of the ascending aorta, revealing marked circumferential wall thickening with a firm, fibrotic, cartilaginous consistency. **(D)** Resected supracoronary ascending aorta in cross-section, demonstrating pronounced eccentric wall thickening with relative preservation of the luminal surface.

During observation, he developed acute deterioration with severe dyspnea, tachypnea (34 breaths/min), hypoxemia (SpO₂ 87%), and hypotension (67/40 mmHg). Advanced airway management was initiated, and norepinephrine infusion was started. Urgent transthoracic echocardiography (TTE) revealed a large circumferential pericardial effusion [PE] (up to 40 mm), left ventricular ejection fraction of 35%, basal-to-mid septal and anterolateral akinesia, and mild mitral regurgitation. Notably, there was no right-sided chamber collapse, making classic tamponade physiology uncertain (Figures 1A–C). Contrast-enhanced CT was performed, showing mixed-density serohematic pericardial fluid, slightly hyperdense [3–30 Hounsfield units (HU)] without evidence of ventricular rupture (Figure 1D). Non-contrast CT showed a crescent-shaped hyperdense lesion measuring 61 HU extending from the aortic root through the ascending aorta, aortic arch, and descending thoracic aorta, reaching 20 mm distal to the origin of the left subclavian artery (Figure 2A, Supplementary Video 1). A small ulcer-like projection (3 mm in diameter, 2 mm in depth) was identified proximal to the brachiocephalic trunk. Following contrast administration, homogeneous aortic opacification was observed (up to 475 HU), without intimal flap, aneurysmal dilatation, or penetrating atherosclerotic ulcer (Figure 2B, Supplementary Video 2). These findings were interpreted as Stanford type A [DeBakey type I] IMH involving the origin and proximal third of the supra-aortic vessels with extension to the infrarenal aorta (Supplementary Figure 2). Coronary CT angiography additionally demonstrated multivessel disease, including severe LAD (70%–75%), circumflex (70%–99%), and RCA (70%–99%) stenoses.

## Differential diagnosis

The initial differential diagnosis was centered on high-risk NSTEMI, supported by dynamic electrocardiographic changes and rising troponin levels. The presence of a large PE in this context raised concern for mechanical complications of myocardial infarction, including free wall rupture or papillary muscle dysfunction. However, the abrupt onset of severe chest pain followed by rapid hemodynamic deterioration broadened the differential toward AAS, particularly IMH or penetrating aortic ulcer. The subsequent identification of extensive aortic wall thickening on CT further increased suspicion for AAS as the unifying diagnosis. Pericardial disease also became a key consideration after detection of a large circumferential effusion, with concern for hemopericardium and evolving tamponade physiology, although the absence of right-sided chamber collapse made classic tamponade less certain. Pulmonary embolism was additionally considered given the acute onset of dyspnea, hypoxemia, and hypotension. As the clinical course progressed toward shock, the differential diagnosis became increasingly focused on catastrophic structural etiologies, particularly AAS or mechanical complications of myocardial infarction, prompting urgent imaging and surgical evaluation.

## Treatment

An urgent pericardial window was performed, draining 550 mL of serohematic fluid, briefly improving hemodynamics. However, within two hours, the patient deteriorated again, with recurrent hypotension, increasing pericardial drainage (350 mL in 2 h), and escalating vasopressor requirements. Given the rapid clinical progression, a multidisciplinary team—including cardiac intensivists, cardiothoracic surgeons, clinical cardiologists, and cardiovascular imaging specialists—concluded that, despite the absence of overt rupture or contrast extravasation on CT, the overall findings were highly suggestive of a contained rupture of the ascending aorta in the setting of an extensive IMH. In this context, emergent surgical intervention was indicated despite prohibitive operative risk.

Intraoperatively, the pericardium was found to be densely infiltrated and filled with organized clot. The ascending aorta was markedly thickened, indurated, and “cartilaginous”, with extension into the aortic arch (Figures 2C,D). After cross-clamping and aortotomy, the luminal surface was unexpectedly normal, without evidence of IMH, dissection flap, or intimal disruption. A supracoronary ascending aortic replacement with a 22-mm Dacron graft was performed. Concomitant coronary artery bypass grafting was undertaken using the left internal mammary artery to the LAD and a reversed saphenous vein to the obtuse marginal branch. Due to extensive involvement of the LAD, endarterectomy with removal of a previously implanted metallic stent was required. Despite maximal surgical and hemodynamic support, the patient developed uncontrollable bleeding, new extensive inferior myocardial infarction, and progressive cardiac failure.

## Outcome and follow-up

The patient progressed to refractory cardiogenic shock with subsequent cardiac arrest despite maximal mechanical and pharmacologic support and died intraoperatively. At the time of death, the underlying etiology of the clinical presentation remained unclear.

**Figure 3.**
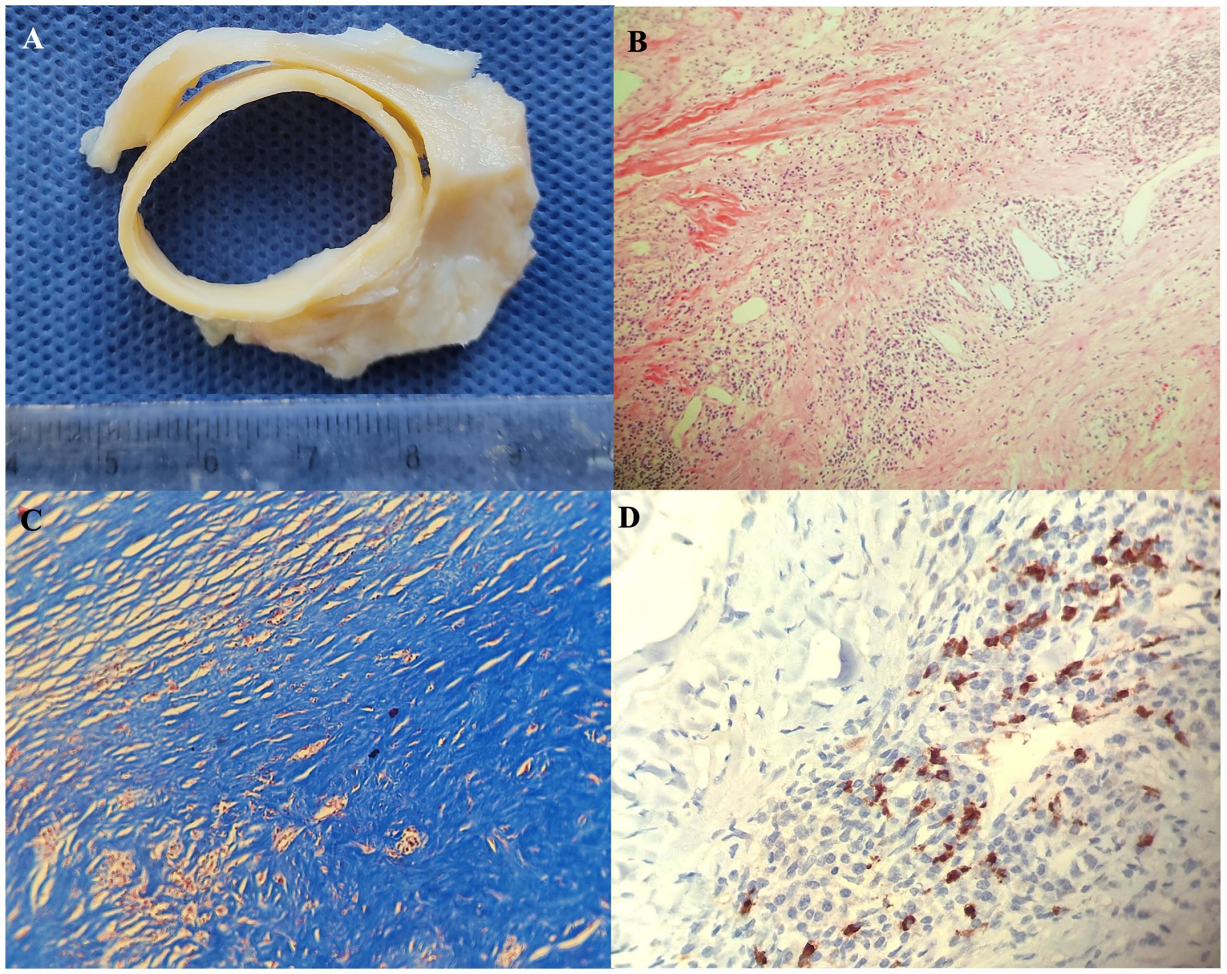
**Pathologic confirmation of IgG4-related aortitis. (A).** Gross specimen showing a transverse section of the ascending aorta with marked eccentric wall thickening (12 mm). **(B).** Hematoxylin–eosin stain (10×) demonstrating a dense perivascular lymphoplasmacytic infiltrate. **(C).** Masson trichrome stain (10×) highlighting extensive fibrotic matrix deposition (blue). (**D.**) Immunohistochemistry (10×) showing IgG4-positive plasma cells, accounting for approximately 30% of inflammatory cells.

Resected aortic tissue was therefore submitted for histopathological analysis. Several weeks later, examination revealed dense lymphoplasmacytic infiltration rich in IgG4-positive plasma cells, along with storiform fibrosis, establishing the diagnosis of IgG4-related aortitis (Figure 3). This etiology had not been suspected clinically, radiographically, or intraoperatively.

## Discussion

IgG4-related aortitis represents an under-recognized form of large-vessel inflammatory disease that may present with acute cardiovascular manifestations indistinguishable from structural aortic pathology (7, 9, 10). In this context, the principal challenge lies not in defining the disease, but in recognizing it during time-critical diagnostic pathways designed for AAS (1, 8). Histopathological confirmation remains the diagnostic cornerstone, requiring >10–30 IgG4-positive plasma cells per high-power field and an IgG4/IgG ratio >40%, often accompanied by storiform fibrosis (2, 8, 11). Although serum IgG4 levels may support the diagnosis, they are normal in up to 30%–40% of biopsy-proven cases (12), limiting their utility in acute presentations (1, 8). In emergent scenarios such as the present case, histopathology ultimately remains the gold standard.

Within this diagnostic framework, a central finding of our case is the ability of IgG4-related aortitis to closely mimic IMH due to significant radiologic overlap (6, 8, 12). In our patient, non-contrast CT demonstrated a crescent-shaped hyperdense lesion measuring 61 HU, extending from the aortic root through the ascending aorta while contrast-enhanced imaging showed homogeneous aortic opacification increased up to 475 HU, without evidence of intimal flap, aneurysmal dilatation, or penetrating atherosclerotic ulcer. Although IgG4-related aortitis typically presents with lower attenuation values (30–50 HU) and variable enhancement (60–100 HU) (13, 14), and IMH with higher non-contrast attenuation (50–70 HU) without enhancement and a characteristic crescent-shaped wall thickening (“crescent sign”) (15–17), these features frequently overlap in acute settings. In this case, the crescentic pattern and initial attenuation strongly favored IMH, illustrating how imaging interpretation is shaped by clinical context and cardiovascular bias, ultimately leading to exclusion of inflammatory etiologies during the acute evaluation.

This diagnostic trajectory is consistent with previously reported cases, in which IgG4-related aortitis is rarely suspected at presentation. Most reports describe alternative initial diagnoses, including anterior mediastinal tumors such as lymphoma, sarcoma, or thymoma (18), as well as inflammatory vasculitides such as giant cell arteritis, Takayasu arteritis, and Behçet disease. In some cases, IMH or aortic dissection were initially diagnosed (12, 19). Overall, the number of reported cases remains limited. Clinically, persistent chest or abdominal pain is reported in approximately 20% of patients (12) and often evolves insidiously over months, as in our patient, while other symptoms such as cough (65%) and dyspnea (30%) are nonspecific (9). Laboratory findings similarly lack specificity (6); elevations in inflammatory markers, troponins, and natriuretic peptides frequently reinforce the suspicion of acute coronary or aortic syndromes. In our case, this diagnostic pathway was further influenced by established cardiovascular risk factors, including male sex, prior smoking exposure, previous coronary disease, and advanced age – features that, although also associated with IgG4-RD (9, 11), are typically recognized only retrospectively.

Given this presentation, management was guided by established recommendations for AAS. The diagnosis of Stanford type A IMH with hemodynamic instability prompted urgent surgical intervention in accordance with ESC and ACC/AHA guidelines (Class I-C and I-B-NR, respectively) (12, 17, 20), supported by registry data demonstrating mortality rates of up to 40% with nonoperative management (5, 7). This approach mirrors previously reported cases in which surgery was undertaken without suspicion of underlying inflammatory disease. However, once IgG4-RD is identified, management differs fundamentally, as glucocorticoids represent first-line therapy and are associated with rapid clinical and radiologic improvement in 70%–90% of patients and reduced risk of aneurysm progression when treated early (8, 11). In our case, intraoperative findings of dense, “cartilaginous” inflammatory tissue involving the coronary vasculature posed significant technical challenges. Extensive LAD endarterectomy with stent removal was performed in the setting of profound hemodynamic instability and diagnostic uncertainty. It is plausible that manipulation of severely inflamed tissue contributed to diffuse coagulopathic bleeding and adverse outcomes, highlighting the risks of aggressive coronary intervention in unrecognized systemic inflammatory aortopathy and emphasizing the importance of intraoperative adaptability.

A key pathophysiological insight of this case relates to the presence of a large sero-hemorrhagic pericardial effusion, a finding not consistently reported in prior cases. Initially interpreted as a complication of myocardial infarction or aortic rupture, this was discordant with intraoperative inspection, which revealed a normal luminal surface without dissection or IMH. In classical IMH, PE results from blood transudation through a weakened adventitia from medial hemorrhage. In the absence of intramural blood, an alternative mechanism must be considered. IgG4-RD provides a plausible explanation, as dense adventitial inflammation and periaortitis may lead to exudative or hemorrhagic effusion through vasa vasorum disruption and inflammatory breach of the adventitial barrier, independent of an intimal tear (9, 12). Additionally, IgG4-mediated pericardial involvement (characterized by lymphoplasmacytic infiltration, obliterative phlebitis, and increased microvascular permeability) can result in erythrocyte extravasation and a sero-hemorrhagic profile (21–23). This mechanism explains the apparent discrepancy between imaging and surgical findings and clarifies how the combination of aortic wall thickening and PE closely mimicked IMH despite a purely inflammatory process demonstrated by histopathology.

This case also highlights important diagnostic limitations. Serum IgG4 levels were not measured during the acute evaluation, and systematic assessment for extravascular involvement was not performed. Retrospective imaging review identified mediastinal lymph nodes up to 11 mm without definitive findings and no clear involvement of other typical organs such as the pancreas, biliary tract, salivary glands, lacrimal glands, or retroperitoneum (11). Importantly, neither elevated serum IgG4 levels nor extravascular manifestations are mandatory for diagnosis (24). In this context, IgG4-related aortitis remains largely unrecognized within current diagnostic pathways (6, 11, 12), as existing ESC and ACC guidelines for AAS do not incorporate inflammatory aortitides, and consensus statements on IgG4-RD offer limited guidance for acute thoracic aortic presentations (8). Consequently, many patients are managed as AAS, with the correct diagnosis established only retrospectively, after surgical or postmortem histopathologic analysis (6, 24). Collectively, this gap suggests that IgG4-related aortitis may be underdiagnosed rather than truly rare (12, 24), remaining largely invisible within guideline-directed clinical decision-making despite its capacity to closely mimic catastrophic cardiovascular emergencies (11).

This case underscores the importance of maintaining diagnostic vigilance in ambiguous presentations and highlights the value of multidisciplinary evaluation and multimodality imaging in identifying non-classical aortic pathology (12). The rare involvement of the ascending aorta, combined with the simultaneous mimicry of acute coronary and aortic syndromes, emphasizes the diagnostic complexity of this entity. Ultimately, the definitive diagnosis relied on histopathology, supported by comprehensive imaging and intraoperative correlation. By illustrating this critical diagnostic blind spot, the present report aims not only to expand the existing literature but also to prompt greater clinical awareness, encouraging earlier consideration of inflammatory aortopathies in patients with discordant clinical and imaging findings, and fostering further investigation into this under-recognized disease.

## Take-away lessons

- IgG4-related aortitis can mimic both acute aortic or coronary syndromes, leading to high-risk diagnostic misclassification in patients with acute chest pain.
- Ascending aortic involvement is rare and often indistinguishable from acute aortic syndromes on initial evaluation.
- Multimodality imaging is essential but may be insufficient to differentiate inflammatory from structural aortic pathology, especially when radiologic features overlap.
- Histopathology remains the definitive method for diagnosis, particularly in unstable or fatal presentations.

## Patient perspective

Due to the fulminant clinical course and intraoperative death of the patient, it was not possible to obtain a patient perspective.

## Patient consent statement

The patient is deceased. Despite reasonable efforts, consent from the next of kin could not be obtained. This report contains no identifiable personal data, and all clinical information and images have been fully anonymized. The authors believe that publication of this case has substantial educational value and is consistent with ethical standards and the principles of the Declaration of Helsinki

## Data Availability

The original contributions presented in the study are included in the article/Supplementary Material, further inquiries can be directed to the corresponding author.
