# Supplementary figures and images for: IgG4-related aortitis mimicking acute aortic and coronary syndromes, multimodality imaging–pathology correlation: a case report
Source: Front Cardiovasc Med. 2026 Jun 4;13:1819638. doi: 10.3389/fcvm.2026.1819638 (PMC13275671; doi:10.3389/fcvm.2026.1819638)

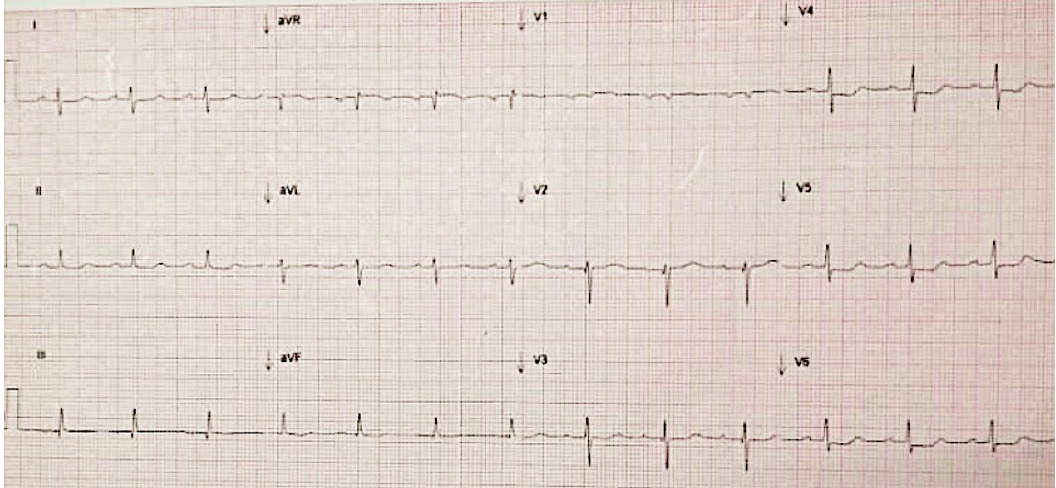

Supplement: Supplementary file 3 [file Image1.jpeg]

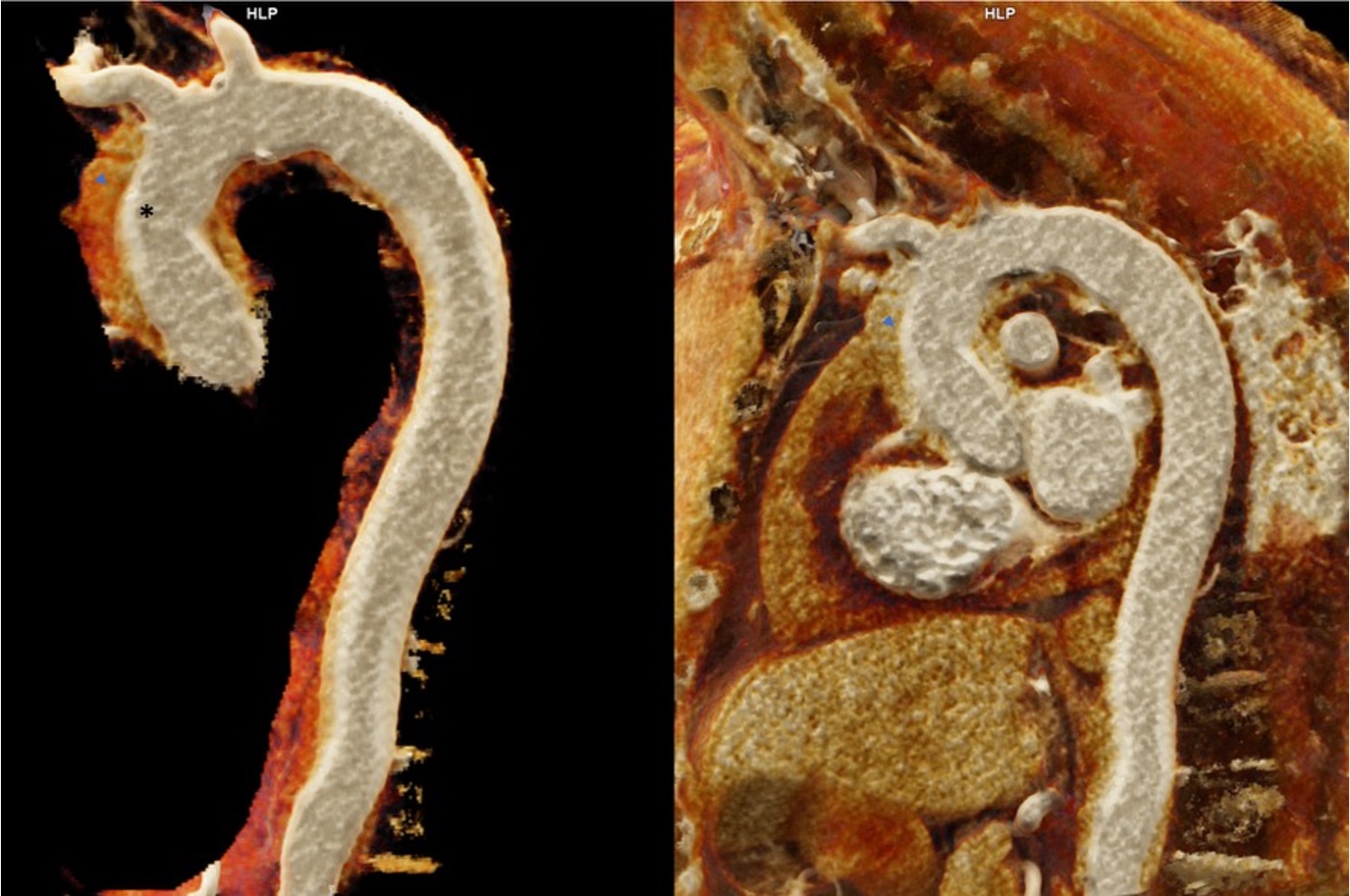

Supplement: Supplementary file 4 [file Image2.jpeg]
